# Sugar perception in honeybees

**DOI:** 10.3389/fphys.2022.1089669

**Published:** 2023-01-13

**Authors:** Laura Değirmenci, Fabio Luiz Rogé Ferreira, Adrian Vukosavljevic, Cornelia Heindl, Alexander Keller, Dietmar Geiger, Ricarda Scheiner

**Affiliations:** ^1^ Behavioral Physiology and Sociobiology, Biocenter, Julius-Maximilians-Universität Würzburg, Wuerzburg, Germany; ^2^ Molecular Plant Physiology and Biophysics, Julius-von-Sachs-Institute, Julius-Maximilians-Universität Würzburg, Wuerzburg, Germany; ^3^ Organismic and Cellular Interactions, Faculty of Biology, Ludwig-Maximilians-Universität München, Munich, Germany

**Keywords:** AmGr1, AmGr2, AmGr3, *Xenopus* oocytes, sugar responsiveness, proboscis extension response (PER), gustatory receptors (Grs), honeybee taste perception

## Abstract

Honeybees (*Apis mellifera*) need their fine sense of taste to evaluate nectar and pollen sources. Gustatory receptors (Grs) translate taste signals into electrical responses. *In vivo* experiments have demonstrated collective responses of the whole Gr-set. We here disentangle the contributions of all three honeybee sugar receptors (AmGr1-3), combining CRISPR/Cas9 mediated genetic knock-out, electrophysiology and behaviour. We show an expanded sugar spectrum of the AmGr1 receptor. Mutants lacking AmGr1 have a reduced response to sucrose and glucose but not to fructose. AmGr2 solely acts as co-receptor of AmGr1 but not of AmGr3, as we show by electrophysiology and using bimolecular fluorescence complementation. Our results show for the first time that AmGr2 is indeed a functional receptor on its own. Intriguingly, AmGr2 mutants still display a wildtype-like sugar taste. AmGr3 is a specific fructose receptor and is not modulated by a co-receptor. Eliminating AmGr3 while preserving AmGr1 and AmGr2 abolishes the perception of fructose but not of sucrose. Our comprehensive study on the functions of AmGr1, AmGr2 and AmGr3 in honeybees is the first to combine investigations on sugar perception at the receptor level and simultaneously *in vivo*. We show that honeybees rely on two gustatory receptors to sense all relevant sugars.

## Introduction

Honeybees depend on floral nectars and honeydew as carbohydrate sources. These comprise the sugars sucrose, glucose, fructose, melezitose and small amounts of other sugars (Ball, 2007; Pita-Calvo and Vázquez, 2017). Honeybees prefer these sugars as well as maltose and trehalose when foraging (Wykes, 1952; Graham, 1992; Roces and Blatt, 1999; Chalcoff et al., 2006; Stanley et al., 2013; Bertazzini and Forlani, 2016; Ryniewicz et al., 2020). Honeybees have only 10 Gr genes in their genome (Robertson and Wanner, 2006; Robertson and Wanner, 2006; Simcock et al., 2017). This number is very low compared to other insects such as the fruit fly (*Drosophila melanogaster*) with 68 genes and the mosquito (*Anopheles gambiae*) with 76 genes. With three Gr genes recognizing sugars, the honeybee has a comparatively reduced set of receptors for the yet broad sugar spectrum. So far, it has remained unknown how these receptors AmGr1, AmGr2, AmGr3 interact with each other. The sugar receptors are located on the antennal tips, the pre-tarsi and the mouthparts of the honeybees, but also internally in brain and gut (Haupt, 2007; Simcock et al., 2017). Honeybees possess one specific fructose receptor (AmGr3; Takada et al., 2018; Değirmenci et al., 2020) and one broadly tuned receptor (AmGr1) detecting sugars such as sucrose, glucose, trehalose and maltose (Jung et al., 2015). The third receptor, AmGr2, has been assumed to act as co-receptor of AmGr1 (Jung et al., 2015; Takada et al., 2018; Değirmenci et al., 2020). Based on the taste range of honeybees and the structural similarity of many sugar molecules, AmGr1 and AmGr2 (as co-receptor) might also respond to other sugars, but we are far from understanding the interaction of these receptors and require conclusive co-expression analyses. We assume that AmGr1 is capable of detecting many more sugars in the taste spectrum of honeybees than have been reported so far by interacting with AmGr2 as its co-receptor. With respect to the seven other putative Grs, only AmGr10 —assumedly a broad amino acid sensing receptor– has been characterized recently (Lim et al., 2019).

We here combine a refined electrophysiological analysis of honeybee gustatory receptors heterologously expressed with genetic knock-out of individual receptors and behavioural analysis of bees. Our data reveal new insight into the interaction of the three sugar receptors in honeybees and can explain the discrepancy between comparatively low number of sugar receptors and a broad taste spectrum in this insect.

## Results

### AmGr1 is essential for sucrose and glucose perception *in vivo* and when expressed heterologously

**FIGURE 1:**
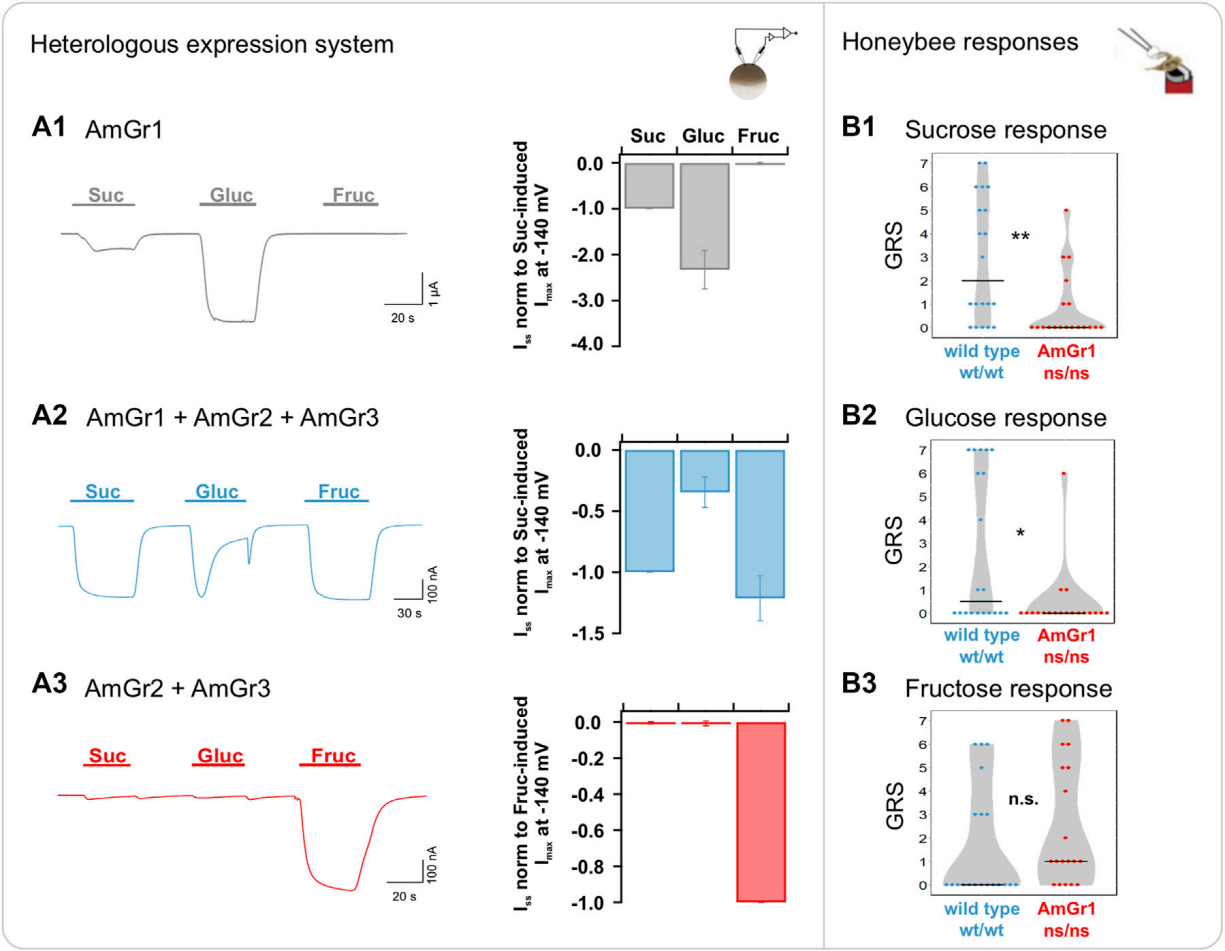
Functional analysis of *A. mellifera* gustatory receptor AmGr1 using a matched heterologous expression system and *in vivo* comparative approach. **(A1–A3)**: two-electrode voltage clamp measurements: Current traces were recorded at a holding potential of −80 mV in response to perfusion with sucrose (Suc), glucose (Gluc) and fructose (Fruc) in standard solution (left panel). Sugar-induced steady-state currents (*I*_*SS*_) were recorded at a membrane potential of −140 mV (middle panel). **(A1)** Representative whole oocyte current trace of AmGr1-expressing oocyte (left panel); Quantification of sugar-induced *I*_*SS*_ *.* Currents were normalized to sucrose-evoked *I*_*SS*_ at −140 mV (mean of *n* = 16 oocytes ± SD; middle panel). **(A2)** Control: Inward whole oocyte currents from AmGr1/AmGr2/AmGr3-expressing oocyte (wild-type mimicry; left panel); Quantification of sugar-induced *I*_*SS*_ that were normalized to sucrose-evoked *I*_*SS*_ at −140 mV (mean of *n* = 10 oocytes ± SD, middle panel). These same values are displayed in all figures as consistent control. **(A3)** Whole oocyte currents from AmGr2/AmGr3 co-expressing oocyte (left panel); Quantification of sugar-induced *I*_*SS*_ *.* Currents are normalized to the fructose-evoked *I*_*SS*_ at −140 mV (mean of *n* = 9 oocytes ± SD; middle panel). **(B1–3)**: behavioural evaluation through proboscis extension response (PER, *in vivo*). Wild-type (wt/wt, *N* = 20) and AmGr1 mutant bees (ns/ns; *N* = 19) were presented a series of sugar concentrations (16%, 20%, 25%, 32%, 40%, 50% and 63% (*w/v*)*;* representing .47 M, .58 M, .73 M, .93 M, 1.17 M, 1.46 M and 1.84 M) of all three sugars sucrose **(B1)**, glucose **(B2)** and fructose **(B3)**. The sum of the responses (PERs) towards the concentrations of one of the sugars was recorded as a sugar-specific GRS (gustatory response score). The distribution of all GRS values of all measured bees is shown as data points and the resulting medians as lines. AmGr1 mutants were less responsive to sucrose when compared with wild-type bees and had significantly lower GRS **(B1)**; Mann-Whitney-U, ns/ns vs. wt/wt, *p* = .0032, **). Glucose responsiveness in AmGr1 mutants was significantly lower than that in wild-types **(B2)**; Mann-Whitney-U, ns/ns vs. wt/wt, *p* = .0125, *). Both groups did not differ in fructose GRS **(B3)**; Mann-Whitney-U, ns/ns vs. wt/wt, *p* = .0779, n.s.).

Our two-electrode voltage-clamp (TEVC) measurements confirm AmGr1 as a receptor for sucrose and glucose, but not fructose, when transiently expressed in *Xenopus* oocytes (Figure 1A1). Oocytes co-expressing all three sugar receptors (AmGr1-3, representing a wildtype-like set of sugar receptors) elicited sustained inward currents of several nano amps when flushed with sucrose and fructose, whereas currents elicited during glucose application displayed a differed trace shape (Figure 1A2). Although glucose was present for 60 s, the glucose-induced currents appeared only transiently for about 20 s (see below). To verify the impact of AmGr1 on overall sugar responses, co-expression of only AmGr2 and AmGr3 in oocytes was tested, simulating honeybee mutants lacking AmGr1. Under this scenario, only fructose-induced macroscopic currents occurred (Figure 1A3), indicating that AmGr2 does not modulate the fructose-specific receptor AmGr3 in *Xenopus* oocytes. Our behavioural assay revealed that mutant bees lacking a functional AmGr1 were significantly less responsive to sucrose and glucose than wildtype bees (Figure 1B1, B2). However, their responses to fructose did not differ from those of wildtypes (Figure 1B3).

### AmGr2 is a functional receptor and operates as a co-receptor for sucrose and glucose perception

**FIGURE 2:**
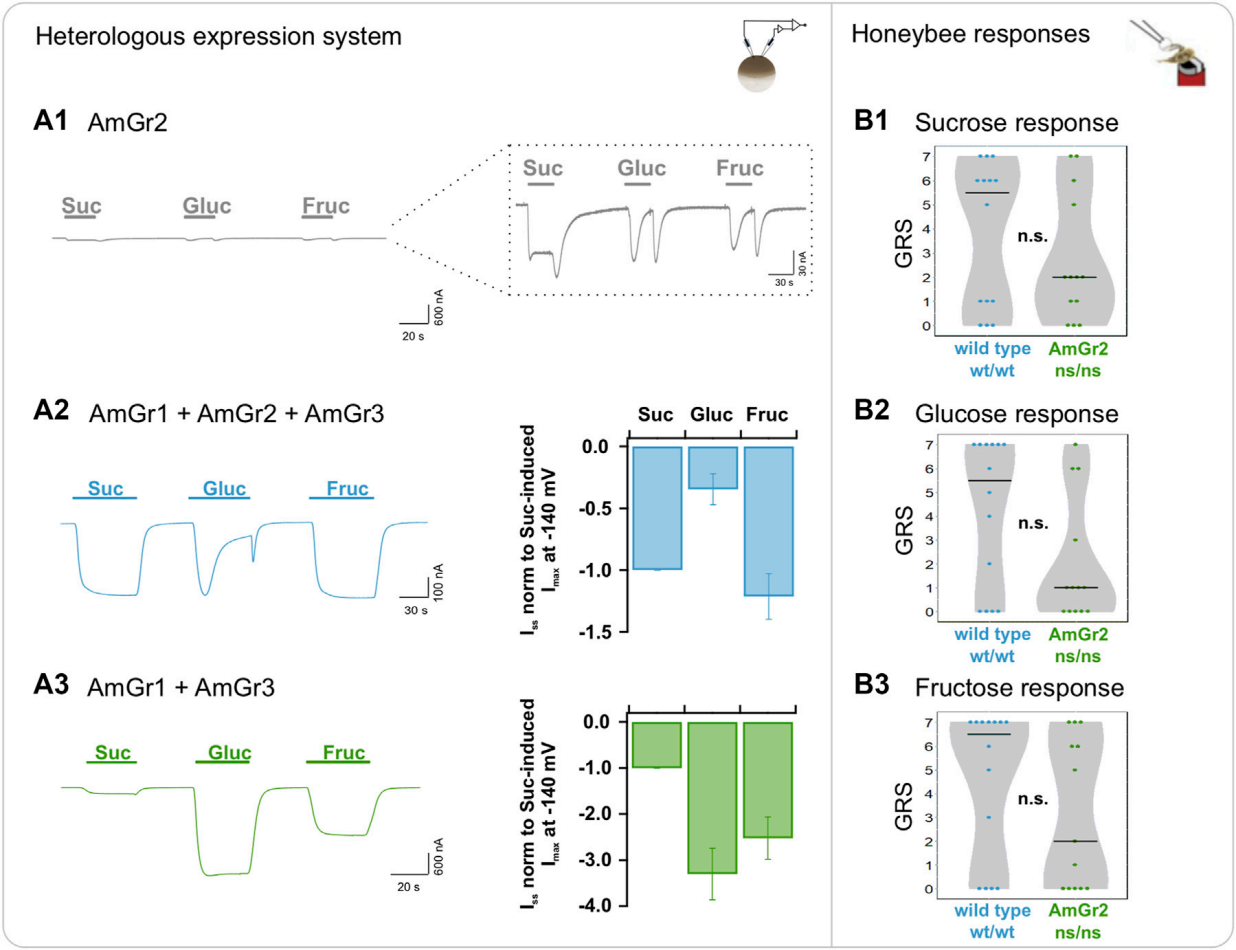
Functional analysis of *A. mellifera* gustatory receptor AmGr2 using a matched heterologous expression system and *in vivo* comparative approach. **(A1–A3)**: two-electrode voltage clamp measurements: Current traces were recorded at a holding potential of −80 mV in response to perfusion with sucrose (Suc), glucose (Gluc) and fructose (Fruc) in standard solution (left panel). Sugar-induced *I*_*SS*_ were recorded at a membrane potential of −140 mV and normalized to the currents in sucrose solution (middle panel). **(A1)** Representative whole oocyte current trace of AmGr2-expressing oocyte. Inset: Magnification of the current trace reveals microscopic sustained or transient inward currents upon sugar application. **(A2)** Control (for clarification displayed again): The inward whole oocyte currents from AmGr1/AmGr2/AmGr3-expressing oocyte (wild type mimicry, left panel); Quantification of sugar-induced *I*_*SS*_ that were normalized to sucrose-evoked *I*_*SS*_ at −140 mV (mean of *n* = 10 oocytes ± SD; middle panel). These same values are displayed in all figures as consistent control. **(A3)** Whole oocyte currents from AmGr1/AmGr3 co-expressing oocyte (left panel); Currents are normalized to the sucrose-evoked *I*_*SS*_ at −140 mV (mean of *n* = 13 oocytes ± SD; middle panel). **(B1–B3)**: behavioural evaluation through proboscis extension response (PER, *in vivo*). Wild-type (wt/wt, *N* = 14) and AmGr2 mutant bees (ns/ns; *N* = 13) were presented a series of sugar concentrations (16%, 20%, 25%, 32%, 40%, 50% and 63% (*w/v*); representing .47 M, .58 M, .73 M, .93 M, 1.17 M, 1.46 M and 1.84 M) of all three sugars sucrose **(B1)**, glucose **(B2)** and fructose **(B3)**. The sum of the responses (PERs) towards the concentrations of one of the sugars was recorded as a sugar-specific GRS (gustatory response score) of each respective bee. The distribution of all GRS values of all measured bees is shown as data points and the resulting medians as lines. AmGr2 mutants (ns/ns) did not show any significant differences in their responsiveness towards all three sugars when compared to wild-type (wt/wt) bees, neither to sucrose **(B1)**; Mann-Whitney-U, ns/ns vs. wt/wt, *p* = .5351, n.s.), to glucose **(B2)**; Mann-Whitney-U, ns/ns vs. wt/wt, *p* = .0909, n.s.) or to fructose **(B3)**; Mann-Whitney-U, ns/ns vs. wt/wt, *p* = .2536, n.s.).

Expression of AmGr2 in oocytes was confirmed by an N-terminal fused YFP as a genetically encoded reporter protein (Supplementary Figure S3A). Intriguingly, AmGr2-expressing oocytes did not reveal any macroscopic sugar-induced currents in TEVC (Figure 2A1). However, reproducible microscopic inward current deflections in response to sugar application –albeit very low– were recorded and reached current amplitudes in the range of tens of nano amps overall (Figure 2A1, inset). Sustained inward currents could only be generated by application of sucrose but not with other sugars. Analogous to the glucose-induced current responses in the wildtype simulation in oocytes (Figure 2A2), AmGr2-expression revealed transient inward currents during glucose application followed by a rapid remission to the baseline. The peak amplitude of the currents lay in the same range as those recorded for sucrose. A similar transient current deflection was observed upon washout with reference solution. Perfusion with fructose evoked a comparable current response pattern to that of AmGr3-expressing oocytes either alone or in combination with AmGr1 and/or AmGr2. Bimolecular fluorescence complementation (BiFC) confirmed physical interaction of AmGr2 subunits, indicating the assembly of homomeric AmGr2 receptors of low electric activity (Supplementary Figure S3B). Taken together, the sugar-induced inward currents, along with the physical interaction between AmGr2 subunits proven with BiFC suggest that AmGr2 assembles to a functional homomeric channel building up an ion pore, thus being able to perform ligand-gated channel activity with low conductance in oocytes by itself. Sucrose and glucose stimulation of oocytes co-expressing AmGr1 and AmGr3 led to a similar current pattern as that seen for the sole expression of AmGr1, with additional fructose-induced currents upon fructose application (Figure 2A3). Honeybee AmGr2-mutants did not differ from wildtype bees in their responses to sucrose, glucose or fructose (Figures 2B1–B3).

### AmGr3 is a specific fructose receptor

**FIGURE 3:**
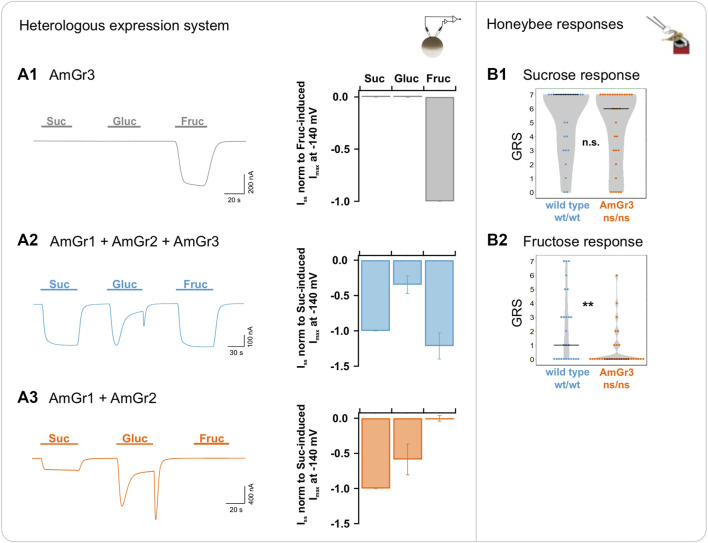
Functional analysis of *A. mellifera* gustatory receptor AmGr3 using a matched heterologous expression system and *in vivo* comparative approach. **(A1–A3)**: two-electrode voltage clamp measurements: Current traces were recorded at a holding potential of −80 mV in response to perfusion with sucrose (Suc), glucose (Gluc) and fructose (Fruc) in standard solution (left panels). Sugar-induced *I*_*SS*_ were recorded at a membrane potential of −140 mV (middle panels). **(A1)** Representative whole oocyte current trace of AmGr3-expressing oocyte (left panel); Currents are normalized to the fructose-evoked *I*_*SS*_ at −140 mV (mean of *n* = 8 oocytes ± SD; middle panel). **(A2)** Control (for clarification displayed again): Inward whole oocyte currents from AmGr1/AmGr2/AmGr3-expressing oocyte (wild-type mimicry, left panel); currents were normalized to sucrose-induced *I*_*SS*_ at −140 mV (mean of *n* = 10 oocytes ± SD; middle panel). These same values are displayed in all figures as consistent control. **(A3)** Whole oocyte currents from AmGr1/AmGr2 co-expressing oocyte (, left panel); currents were normalized to sucrose-induced *I*_*SS*_ at −140 mV (mean of *n* = 13 oocytes ± SD; middle panel). **(B1–B2)** (as published previously in Degirmenci et al., 2020): behavioural evaluation through proboscis extension response (PER, *in vivo*). Wild-type (wt/wt, *N* = 26) and AmGr3 mutant bees (ns/ns; *N* = 31) were presented a series of sugar concentrations (16%, 20%, 25%, 32%, 40%, 50% and 63% (*w/v*); representing .47 M, .58 M, .73 M, .93 M, 1.17 M, 1.46 M and 1.84 M) of the sugars sucrose **(B1)** and fructose **(B2)**. The sum of the responses (PERs) towards one of the sugars was recorded as a sugar-specific GRS (gustatory response score). The distribution of all GRS values of all measured bees is shown as data points and the resulting medians as lines. AmGr3 mutants did not show any difference in GRS when compared with wild-type bees **(B1)**; Mann-Whitney-U, ns/ns vs. wt/wt, *p* = .4279, n.s.). Fructose GRS of AmGr3 mutants were significantly lower than those of wild-types **(B2)**; Mann-Whitney-U, ns/ns vs. wt/wt, *p* = .0062, **). Because of experimental limitations (such as short survival of the AmGr3 mutants), glucose measurements were not implemented and cannot be pursued retrospectively.

The sole expression of AmGr3 in oocytes revealed only fructose-induced current responses (Figure 3A1). No other sugar acted as ligand of AmGr3. Oocytes co-expressing AmGr1 and AmGr2, simulating the honeybee AmGr3 knock-out mutant, did not elicit any fructose-induced currents in the cells (Figure 3A3). Honeybee AmGr3 homozygous mutants displayed a significantly reduced response to fructose compared to wild-type bees. This behavioural difference was not observed when tested with sucrose. These findings show that AmGr3 is unequivocally a fructose-specific receptor in the honeybee.

### Modulation of sugar-induced signals by receptor co-expression

*Xenopus* oocytes expressing all three receptors showed robust current deflections in TEVC when exposed to sucrose, glucose or fructose. Interestingly, glucose-induced inward currents were of transient nature, showing a decay over the course of application (displayed throughout all figures: Figure 1A2; Figure 2A2; Figure 3A2). Following the decay, glucose-induced *I*_*SS*_ reached similar levels of maltose, trehalose and melezitose (Supplementary Figure S1F, bar diagram). This behaviour is also apparent when AmGr3 is absent (Figure 3A3; Supplementary Figure S1C). However, when expressed alone, AmGr1-elicited glucose currents were stronger and did not decay over time (Figure 1A1). AmGr2 itself did not show macroscopic sugar-induced currents (Figure 2A1) which is well in line with honeybee AmGr2 mutants that did not show significant differences in responses to sucrose, glucose or fructose compared to wild-type bees (Figures 2B1–B3). Moreover, our BiFC experiments indicate a direct physical interaction between AmGr1 and AmGr2 and strongly suggest that heteromerization occurs (Supplementary Figure S3B). Thus, AmGr2 potentially acts exclusively as co-receptor for the sucrose signal of AmGr1, modulating strength and time-dependent characteristics of glucose-induced signals.

For fructose-induced currents by AmGr3 activation we did not see any modulation when co-expressing with the other receptors (Figure 1A3 or Figure 2A3). Nevertheless, a physical interaction with AmGr2 on the protein level seems to be possible, even if it did not modulate the fructose specificity of AmGr3 (Supplementary Figure S3). We did not detect any heteromeric formation with AmGr1 in our BiFC experiments (Supplementary Figure S3). Mutant bees expressing AmGr1 and AmGr3 but lacking AmGr2, as well as those expressing AmGr2 and AmGr3 but lacking AmGr1 did not show any significant differences in their fructose response compared to wild-type bees (Figure 1B3; Figure 2B3 ). Thus, AmGr3 is irreplaceable for fructose perception in honeybees and its electrophysiological properties cannot be modulated by neither AmGr1 nor AmGr2.

## Discussion

Sugar taste plays a critical role when evaluating profitable food sources in terms of concentration and type of sugar in honeybees (de Brito Sanchez et al., 2007), since they rely on nectar as their main source of carbohydrates. Honeybees only have 10 Gr genes and hardly anything is known about their gustatory perception (Robertson andWanner, 2006; Simcock et al., 2017). Given that honeybees respond to a large variety of sugars, it is interesting that they achieve this with only three candidate sugar receptors AmGr1-3 (Wykes, 1952; Robertson & Wanner, 2006; Simcock et al., 2017). We hypothesized that honeybees rely on a complex interaction of these receptors to identify the different sugars and investigated for the first time Gr interaction using electrophysiology and behavioural assays.

AmGr1 elicited sugar-induced responses to sucrose, glucose, maltose and trehalose (consistent with Jung et al., 2015) when heterologously expressed. Further, our study reveals that AmGr1 is also capable of perceiving melezitose (Supplementary Figure S1). Melezitose is collected from honeydew, making up to 70% of its sugar fraction (Seeburger et al., 2020), rendering it as an alternative food source (Meiners et al., 2017). However, excessive melezitose intake can lead to health problems in bees, including reduced foraging activities, hair loss and necrotic appearances in the midgut (Horn, 1985; Imdorf et al., 1985; Seeburger et al., 2020). The fact that AmGr1-expressing oocytes recognize melezitose similarly to sucrose and glucose suggests that melezitose evokes a positive response by bees. We here propose that honeybees are unable to discriminate between melezitose from beneficial sugars, exposing a health risk under unfavourable foraging conditions (e.g. over-breeding of aphids; Seeburger et al., 2022). This could be a major reason for the occurrence of honeydew flow disease reported by beekeepers (Alfonsus, 1935).

Furthermore, we have shown for the first time that AmGr1 is directly involved in the evaluation of sucrose, glucose (Figure 1) and maltose at the behavioural level but not in the perception of fructose or arabinose (Supplementary Figure S4). Maltose is found in both honeydew and nectars of many plants, whereas arabinose seems to be present only in traces (Wykes, 1952; Manzanares et al., 2011; Akšić et al., 2020). The substrate specificity of AmGr1 for sucrose, glucose, maltose, trehalose and melezitose (rather than arabinose) might reflect the natural occurrence of these sugars in honeybee resources or in its haemolymph sugar. With this promiscuous ligand specificity, AmGr1 is important for taste perception of honeybees, thereby counterbalancing a comparatively small set of Grs. This shows that AmGr1 inheres a ligand cross-reactivity based on sugar ligands with at least one accessible D-glucose unit. Ligand cross-reactivity is also known in other organisms of this receptor family (see review in *Drosophila*: Slone et al., 2007; Freeman et al., 2014). In contrast, we could not detect any responses to the less relevant sugars such as arabinose, mannose or galactose without glucose unit. Additionally, no signal was generated by raffinose since we assume that its critical glucose unit is embedded and difficult to access. Future experiments combining structure-related functional analysis with glucose analogues might provide new insights regarding its sugar stereospecificity.

Our study provides first evidence that AmGr2 forms a functional receptor, though it does not provide sufficient ion channel performance on a comparable scale to AmGr1 or AmGr3. Furthermore, co-expression of AmGr2 and AmGr3 tagged with complementary YFP-halves revealed fluorescence signals (Supplementary Figure S3B), indicating that AmGr2 can potentially form a heteromer with AmGr3. BiFC results must be carefully interpretated, as cases of false-positives have been reported in the literature. Nevertheless, TEVC experiments provided neither a gain nor a loss of function in oocytes co-expressing both receptors, suggesting that AmGr3 is not modulated by AmGr2. Here, co-expressing AmGr2 with AmGr1 displays a clear co-receptor function, that is in contrast to Jung et al. (2015). It tunes the broad sugar perception of AmGr1 into a specific sucrose receptor by drastically affecting glucose-induced signals. This was observed over the course of long sugar applications (current decrease occurs after 10–15 s; Figure 3A3). We assume that a current remission of transient nature might have been overlooked in the study of Jung et al. (2015), because therein sugar applications lasted 10 s overall and no steady-state currents were used for analysis. The inactivation property of AmGr2 only occurs in co-expression with AmGr1, suggesting that the heteromer adopts an altered, yet fine molecular gating mechanism restricting the ion passage when interacting with ligands other than sucrose (substrate-induced inhibition). When we stimulated oocytes longer than 10 s, inactivation occurred at glucose concentrations higher than 50 mM, with larger doses leading to stronger inactivation (Supplementary Figure S2). Thus, a broad spectrum of sugar taste in honeybees can be fine-tuned to a small set of sugars and AmGr1-2 heteromerization broadens their functional diversity (Xicluna et al., 2007; Geiger et al., 2009). Some of the contrasting results of our study and that by Jung et al. (2015) might be related to differences in the protein sequence used for AmGr1 (here: GenBank accession OP546539). While Jung et al. (2015) used hybrids of *Apis mellifera carnica*, *ligustica* and *caucasica* (H. Kwon, personal communication) derived from Korea, our bees were *Apis mellifera carnica* from a German source (Supplementary Table S1).

AmGr2 mutants did not differ in their responses to sucrose, glucose or fructose compared to wild-type bees. Although our behavioural paradigm works excellently for sugar evaluation in honeybees (Değirmenci et al., 2020), it might be rather unspecific for characterizing a co-receptor like AmGr2 in behaviour. In taste tissues of insects there is a variety of different sensory receptors expressed which might produce overlapping stimuli, so that fine-tuning signals from a co-receptor may be blurred in behaviour (Thorne et al., 2004; Amrein, 2016; Miriyala et al., 2018). In contrast, AmGr1 and AmGr3 mutants, which are directly and exclusively responsible for sugar perception, produce clear phenotypes but not a total loss of sugar responsiveness. This indicates that testing behaviour *in vivo* cannot exclude the influence of other interfering stimuli (Geiger et al., 2009). For instance, it was shown that fixation influences behavioural responses to sugar by inducing stress (Pankiw and Page, 2003). Our electrophysiological results suggest that AmGr2 appears to only act as a co-receptor by modulating sugar signals.

Earlier experiments showed that freely moving or caged bees prefer sucrose over other sugars (von Frisch, 1934; Wykes, 1952; Bachmann and Waller, 1977) comparable to fixed bees in more recent behavioural experiments (Ayestaran et al., 2010; Simcock et al., 2018). In all TEVC experiments, however, the sucrose signals measured were mostly weaker or similar to those of glucose. The yet uncovered co-receptor function of AmGr2 or differences in general receptor expression might thus be factors modulating the receptor signal and the actual behaviour, but these points need further investigation.

Our results prove AmGr3 to be a specific fructose receptor. Cells without this receptor led to an absence of inward currents after applying fructose. Honeybee AmGr3 mutants were significantly less responsive to fructose than wildtypes. For the first time, we can thus prove that AmGr3 is not influenced by any other sugar receptor. Intriguingly, a single receptor seems to be responsible for fructose perception, while AmGr1 detects multiple sugars. We hypothesize that AmGr3 may not only function as a sugar receptor in the peripheral taste perception but may further function as an internal sensor, which is supported by the presence of AmGr3-mRNA in the antennae and brain (Değirmenci et al., 2018). Furthermore, AmGr3 was suggested to detect the nutritional level of haemolymph sugar (Simcock et al., 2017). Levels of haemolymph fructose as well as AmGr3 expression together might orchestrate an intricate mechanism to drive starvation sensation and metabolic responses. Similarly, the receptor homolog of AmGr3 in *Drosophila* (DmGr43a) functions both as receptor and nutrient sensor (Slone et al., 2007; Miyamoto et al., 2012; Freeman et al., 2014; Fujii et al., 2015). Further studies are necessary to precisely unravel the internal role of AmGr3 in the honeybee.

Overall, our matched *in vivo* and functional analyses provide a powerful tool to characterize taste perception at different levels of the system. Thus, the repertoire of sugar taste in honeybees could be expanded and mimicked to full extent of all possible combinations of sugar receptor ensembles in *Xenopus* oocytes. Furthermore, we were able to assign a direct physiological role to these Grs *in vivo*. This approach can be adapted to further uncover taste perception in honeybees, which has been largely ignored due to lack of suitable approaches. It is convincible that honeybees inhere a reduced set of receptors due to co-evolution with plants resulting in a narrow food ecology. Nevertheless, their complex interaction provides an enhanced perception capacity.

Similar to colour vision, which can be achieved by just three photoreceptors (trichromatic vision; Dominy and Lucas, 2001), the broad sugar taste in honeybees can be covered by three sugar receptors. Surprisingly, two receptors (AmGr1 and AmGr3) are sufficient for the basic perception of sugars in honeybees, regardless of the fine-tuning by the co-receptor AmGr2.

## Materials and methods

### RNA extraction and cDNA synthesis

RNA (taste tissue) was extracted according to Değirmenci et al. (2020). RNA was then purified, precipitated, washed, dried and resolved (as described; Değirmenci et al., 2020). The cDNA synthesis was followed by an RNA H digestion. Large scale Phusion PCRs were performed (Supplementary Table S1). PCR products were proven on gel, purified and A-tailed with taq-polymerase as described before (Değirmenci et al., 2020).

### Cloning and cRNA synthesis

PCR fragments were cloned into pGEM-T vector *via* T/A cloning following our previous protocol (Değirmenci et al., 2020). *E. coli* were transfected, selected and cultivated overnight (described in Değirmenci et al., 2020). The plasmid was then isolated and verified through sequencing. Each cDNA was sub-cloned into pNBIu, YFP-fusion and BiFC (Bimolecular Fluorescence Complementation) vectors and respective cRNAs were accomplished using the techniques described in Değirmenci et al. (2020). YFP and complementary YFP-halves were cloned upstream of the respective cDNA (Supplementary Figure S3) and verified by sequencing.

### *Xenopus* oocyte recordings

Oocytes were injected with either 25 ng AmGr1, 50 ng AmGr2 or 50 ng AmGr3 cRNA (sole or co-expression combinations) and incubated for 2–5 days at 16°C in ND96 solution (10 mM HEPES pH 7.4, 96 mM NaCl, 2 mM KCl, 1 mM MgCl_2_, 1 mM CaCl_2_) containing 50 mg/l gentamycin. Electrophysiological experiments were performed using the two-electrode voltage-clamp (TEVC) technique. Standard voltage protocols: holding potential of 0 mV followed by 200 ms voltage pulses (+20 to −140 in 20 mV decrements); single-pulse at −80 mV holding potential. TEVC solutions: 30 mM NaCl, 10 mM Tris/Mes (pH 7.5), 1 mM CaCl_2_, 1 mM MgCl_2_, 2 mM KCl and either 160 mM D-sorbitol (reference solution) or 160 mM of the tested sugars (sucrose, glucose, fructose, maltose, arabinose, mannose, galactose, raffinose, trehalose or melezitose). Sugar-induced steady-state currents (ISS) were derived by subtracting the currents in the absence of sugar from the currents in the presence of sugar and normalized either to sucrose- or fructose-induced *I*_*SS*_ at −140 mV (depending on the AmGr-ensemble).

### Bimolecular fluorescence complementation (BIFC) assay

YFP- or BiFC-derived fluorescence in oocytes were excited with an argon laser line (514 nm) and YFP fluorescence emission was monitored (500–580 nm). Pictures were taken with a confocal laser scanning microscope (Leica TCS SP5; Leica Microsystems GmbH) equipped with a L25×/0.95W objective. Oocytes were injected with 50 ng cRNA of each BiFC construct.

### Preparation of sgRNA

Target-sites for the sgRNAs (single guide RNAs) were found in the first exons of the open reading frames (ORFs) of the respective genes following strict criteria (Supplementary Table S2; Supplementary Figure S6; Değirmenci et al., 2020). The PCR template for sgRNA production was generated with specific primers (for each sgRNA, Supplementary Table S2, Supplementary Figure S6) in an overlapping phusion PCR and purified as described in Değirmenci et al. (2020). According to that work, we produced receptor specific sgRNA for which best hatching and mutation rates were pre-tested. During the experiment, a fresh aliquot of sgRNA and Cas9 enzyme was used per day and stored on ice (concentrations in Supplementary Table S2; protocol of Değirmenci et al., 2020).

### Honeybee egg harvest

The beehives had related and naturally inseminated queens of *Apis mellifera carnica*, which were maintained outdoors at Würzburg University and fed if necessary. Time-monitored eggs were harvested with the JENTER system as described in Değirmenci et al. (2020). Eggs were microinjected 0–1.5 h after deposition (with either sgRNA for AmG1, AmGr2 or AmGr3 and water controls; two replicates; results of replicates in Supplementary Figure S5), assuring mutational events during single-cellular state, leading to fully mutated embryos without mosaic patterns (honeybee zygote division: Yu and Omholt, 1999). This method was proven in our previous work (Değirmenci et al., 2020) and by Roth et al. (2019). Mutations were controlled *via* NGS (next generation sequencing).

### Microinjection of eggs and artificial rearing of honeybees

Following the protocol of Roth et al. (2019), eggs were processed and injected with 400 pl volume (water or sgRNA/Cas9; Supplementary Table S2), using the same conditions, set-up, procedure and material described in our prior work (Schmehl et al., 2016; Değirmenci et al., 2020). Eggs were treated until hatching and the larvae were artificially reared as we described previously (Değirmenci et al., 2020) and based on the protocol of Schmehl et al. (2016). As described, adult bees were individually marked and one wing was removed. All marked bees of one replicate (raised in the same batch, the treatment group with one respective sgRNA/Cas9 and the water control group) were kept in a cage under same conditions described in Değirmenci et al. (2020).

### Testing responsiveness to sugars

All animals tested were raised, kept and tested for sugar responsiveness in the same set-up randomized and with covered marking. Bees were mounted and tested for their proboscis extension response (PER) to increasing concentrations of each of the sugars sucrose, glucose and fructose (alternatingly starting with one of them: 16, 20, 25, 32, 40, 50% and 63% (w/v); representing .47, .58, .73, .93, 1.17, 1.46 and 1.84 M) (Scheiner et al., 2013; Değirmenci et al., 2020). Because of experimental limitations (short survival of the mutants) glucose measurements were not implemented for AmGr3-mutants. Sugar responsiveness is not influenced by the order of concentrations (Scheiner et al., 2013). For each sugar and each bee, the positive PERs towards the concentrations were recorded. The sum of responses to all seven concentrations of a sugar constitutes the individual gustatory response score (GRS, for each sugar) of a bee (Scheiner et al., 2003a; Scheiner et al., 2003b; Scheiner et al., 2004; Scheiner et al., 2013; Değirmenci et al., 2020).

### Genotyping *via* next generation sequencing (NGS)

Honeybee gDNA was isolated as described before (Değirmenci et al., 2020). The gDNA samples of putative mutants and the control group were pre-selected *via* a hex-labelled PCR and fluorescence length analysis (Supplementary Table S2). Subsequently, we performed NGS in multiplex approach with indexed samples as described previously (with GENEWIZ, Leipzig, Germany, Supplementary Table S2; Değirmenci et al., 2020). Sequencing, bioinformatic analysis, demultiplexing, merging of all reads (forward and reverse) and filtering was performed according to Değirmenci et al., in 2020 (Edgar, 2010; Eddy, 2011). Using software and scripts from our previous work, we identified and counted variants of each sample, dereplicated, aligned them with the reference and counted indel positions (Değirmenci et al., 2020).

For AmGr1, the alignment was split into segments to cover only the relevant site to account for splice variants at other positions (Edgar, 2010) before counting indels. Alleles were classified: wild-type as “wt”; in-frame (with indels multiple of 3, intact ORF) as “if”; nonsense (frame shift 1 or 2 leading to a non-functional protein, Supplementary Figure S6) as “ns”. We thus followed the proved genotyping approach of Roth et al. (2019) and only included animals with a proven homozygous mutant (ns/ns) or homozygous wildtype (wt/wt) genotype according to our previous work (Değirmenci et al., 2020).

### Quantification and statistical analysis

At least two independent TEVC experiments (oocytes from different batches) were performed. Sample size *n* and statistical details (mean ± standard deviation, SD) are given in the figure legends. For the behavioural analysis the GRS of mutant and wild-type bees of each sugar were compared using the Mann-Whitney-U test, since data was not normally distributed.

## Data Availability

The datasets presented in this study can be found in online repositories. The names of the repository/repositories and accession number(s) can be found below: https://datadryad.org/stash, https://doi.org/10.5061/dryad.prr4xgxpt.

## Supplementary material

SUPPLEMENTARY FIGURE S1 Representative TEVC recordings of sugar-induced currents derived from *Xenopus* oocytes expressing different Gr-ensembles.

SUPPLEMENTARY FIGURE S2 Glucose dose response of AmGr1 and AmGr1+AmGr2 expressing oocytes.

SUPPLEMENTARY FIGURE S3 Fluorescence-based studies of Xenopus oocytes expressing different YFP-fused AmGr1-3 constructs.

SUPPLEMENTARY FIGURE S4 Behavioural PER test with AmGr1 mutants (ns/ns), half-mutated and half in-frame-mutated bees of AmGr1 (ns/if) and wildtype bees (wt/wt) with a 30% solution of several other sugars (glucose, sucrose, fructose, maltose, arabinose) and water.

SUPPLEMENTARY FIGURE S5 Replicates of behavioural evaluation through proboscis extension response (PER, in vivo) according to the section B of each figure (Figures 1–3).

SUPPLEMENTARY FIGURE S6 Overview of the receptors (AmGr1, AmGr2 and AmGr3) gene sequences and the early stop codons introduced by mutational frameshifts.
