# Supplementary material for: Sugar perception in honeybees
Source: Front Physiol. 2023 Jan 13;13:1089669. doi: 10.3389/fphys.2022.1089669 (PMC9880324; doi:10.3389/fphys.2022.1089669)
Supplement: Supplementary file 1 [file DataSheet1.docx]

**Supplementary Information**

For:

**Sugar perception in honeybees**Laura Değirmenci^†^ & Fabio Luiz Rogé Ferreira^†^, Adrian Vukosavljevic, Cornelia Heindl, Alexander Keller, Dietmar Geiger, Ricarda Scheiner

**The following supporting information is available for this article:**

**Fig. S1: Representative TEVC recordings of sugar-induced currents derived from *Xenopus* oocytes expressing different Gr-ensembles.**

**Fig. S2: Glucose dose response of AmGr1 and AmGr1+AmGr2 expressing oocytes.**

**Fig. S3: Fluorescence-based studies of *Xenopus* oocytes expressing different YFP-fused AmGr1-3 constructs.**

**Fig. S4: Behavioural PER test with AmGr1 mutants (ns/ns), half-mutated and half in-frame-mutated bees of AmGr1 (ns/if) and wildtype bees (wt/wt) with a 30% solution of several other sugars (glucose, sucrose, fructose, maltose, arabinose) and water.**

**Fig. S5: Replicates of behavioural evaluation through proboscis extension response (PER, in vivo) according to the section B of each figure (Fig. 1-3).**

**Figure S6: Overview of the receptors (AmGr1, AmGr2 and AmGr3) gene sequences and the early stop codons introduced by mutational frameshifts.**

**Table S1: Annotations and primers used for cloning the respective AmGr genes into expression vectors.**

**Table S2: Bioinformatic information for performing CRISRP/Cas9 in the genes of the sugar receptors AmGr1, AmGr2 and AmGr3.**


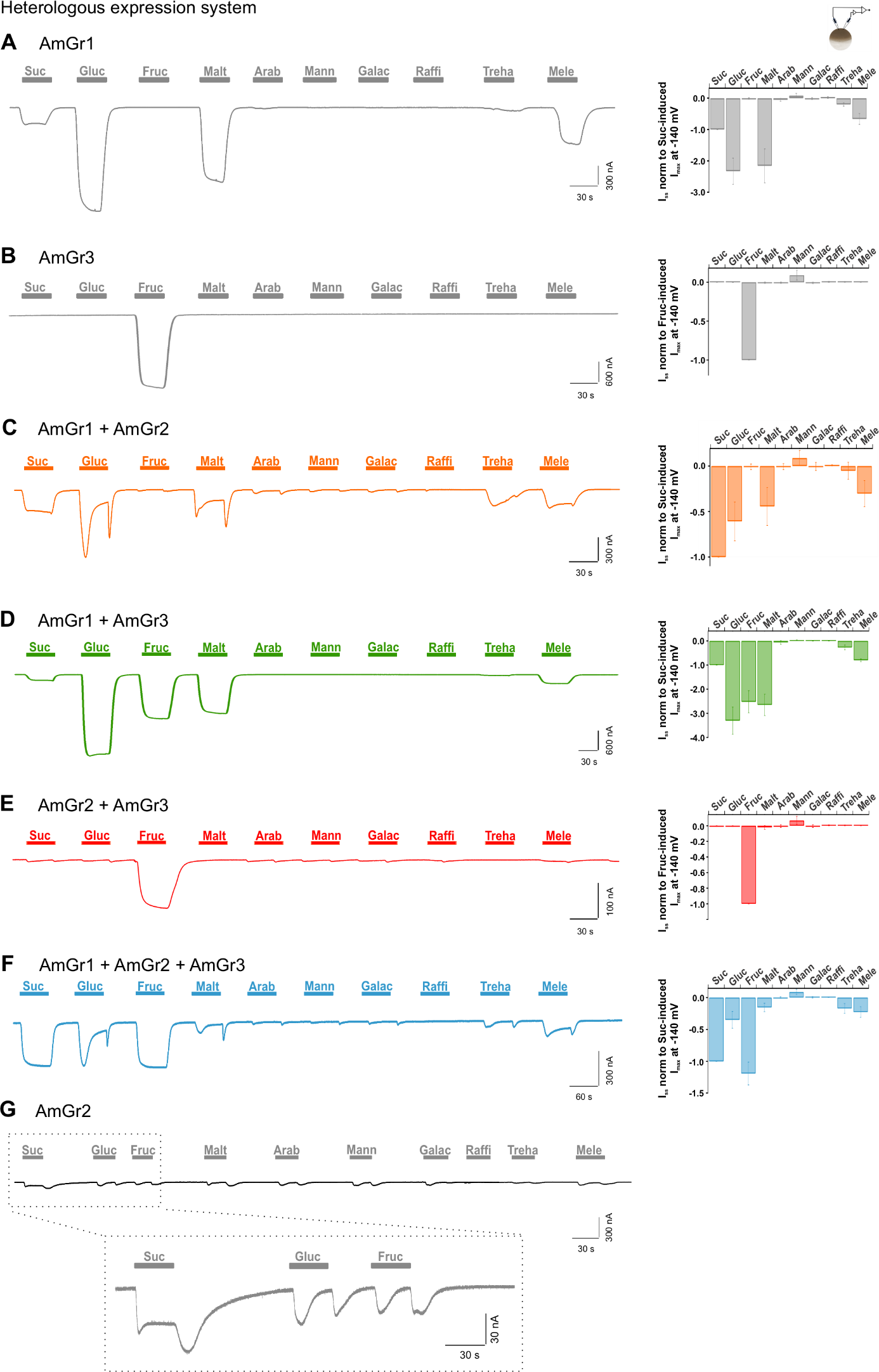


**Figure S1: Representative TEVC recordings of sugar-induced currents derived from *Xenopus* oocytes expressing different Gr-ensembles.** The gustatory receptors AmGr1-3 have been transiently expressed in *Xenopus* oocytes either alone or in different Gr combinations. Oocytes were clamped at a holding membrane potential of -80 mV and tested for sugar response to sucrose (Suc), glucose (Gluc), fructose (Fruc), maltose (Malt), arabinose (Arab), mannose (Mann), galactose (Galac), raffinose (Raffi) and melezitose (Mele) (160 mM each; perfusion indicated by bars). For quantification of sugar specificities, sugar-induced steady-state currents (*I_SS_*) were recorded at a membrane potential of -140 mV and normalized to the currents in either sucrose (A, C, D and F) or fructose (B and E) solution (bar diagrams). **A** AmGr1 (mean of *n* = 16 oocytes ± SD); **B** AmGr3 (mean of *n* = 8 oocytes ± SD); **C** AmGr1 and AmGr2 co-expression (mean of *n* = 13 oocytes ± SD); **D** AmGr1 and AmGr3 co-expression (mean of *n* = 13 oocytes ± SD); **E** AmGr2 and AmGr3 co-expression (mean of *n* = 9 oocytes ± SD); **F** AmGr1, AmGr2 and AmGr3 co-expression (mean of *n* = 10 oocytes ± SD); **G** AmGr2, Inset: magnification of the current trace of AmGr2-expressing oocyte for sucrose, glucose and fructose application.


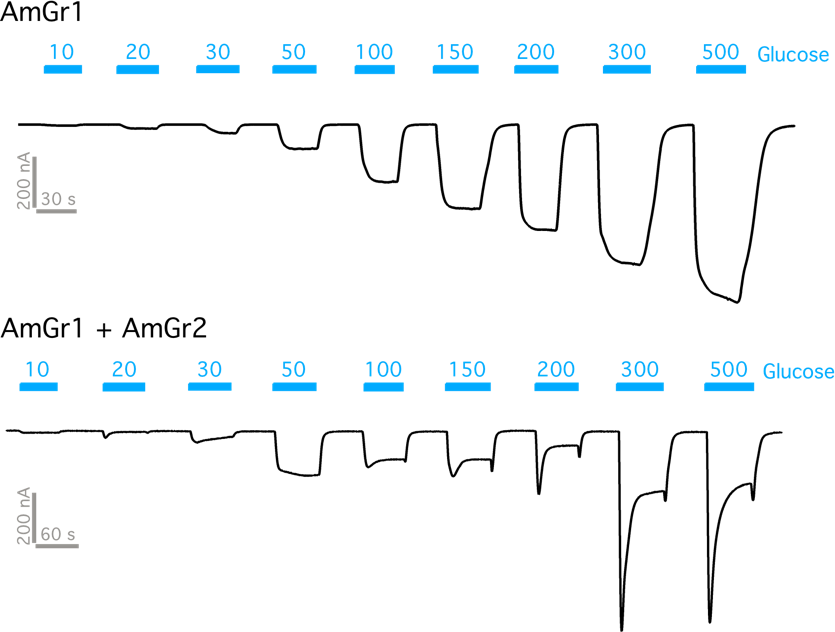


**Figure S2: Glucose dose response of AmGr1 and AmGr1+AmGr2 co-expressing oocytes.** Representative TEVC recordings of glucose-induced current traces elicited by stimulation with different glucose concentrations. Oocytes expressing either AmGr1 alone (upper panel) or co-expressing both AmGr1 and AmGr2 (lower panel) were clamped at -80 mV and subsequently perfused with increasing glucose concentrations (10 – 500 mM). Between each glucose application, reference solution was perfused until glucose-induced currents returned to the pre-stimulus level. Sugar applications for co-expressing oocytes were extended to 60 s to comprehensively track the course of transient currents.


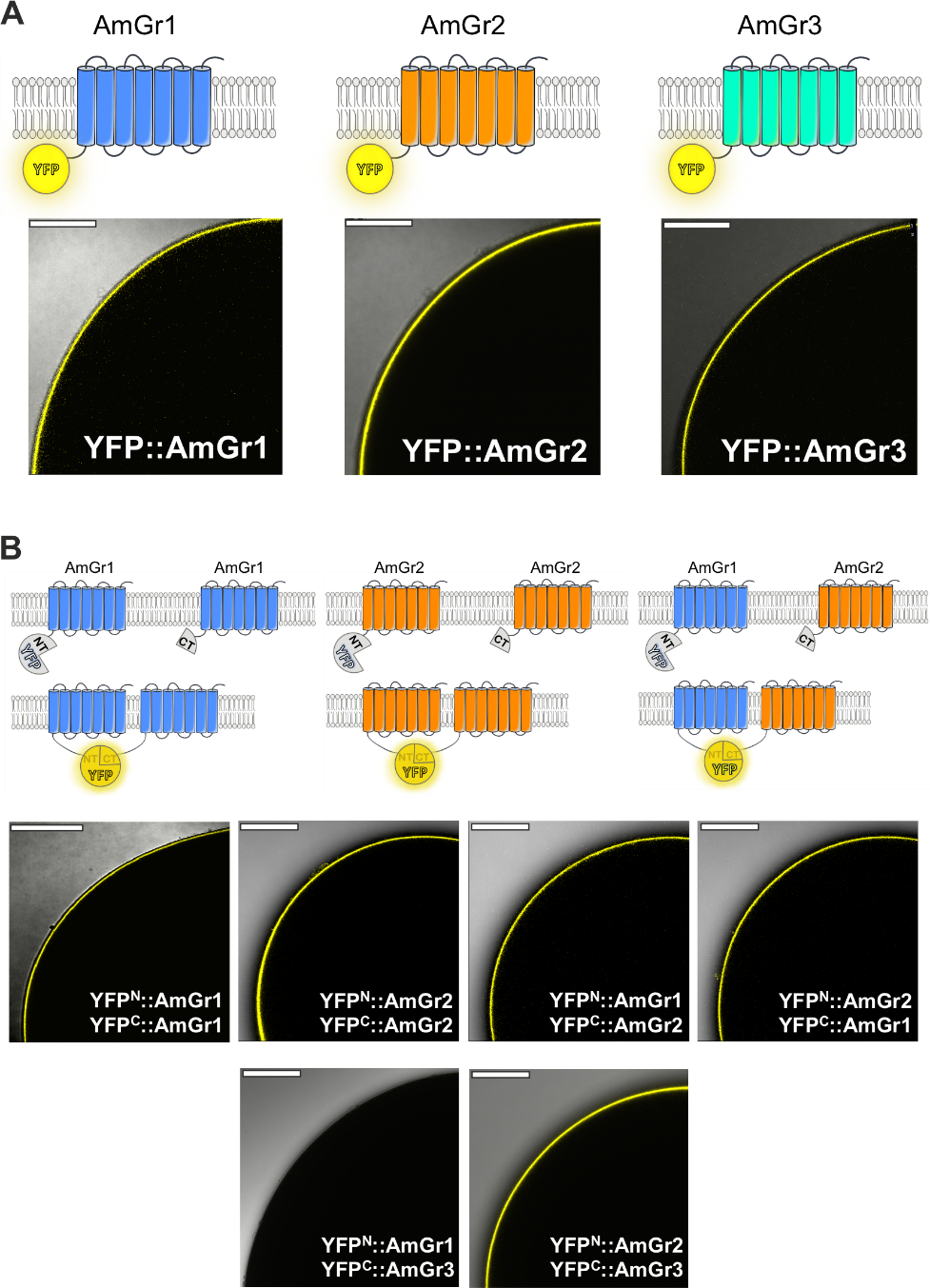


**Figure S3: Fluorescence-based studies of *Xenopus* oocytes expressing YFP-tagged AmGr1-3 constructs.** Schematic models of YFP-tagged AmGrs and AmGrs tagged with YFP halves for BiFC experiments (A and B, upper panels); Pictures show a quarter of an optical slice of an oocyte, representing an overlay of brightfield and detection of fluorescence (depicted in yellow). Images were taken with a confocal laser scanning microscope (A and B, lower panels). **(A)** Representative images of AmGr1‑3 tagged with YFP to the N-terminus (YFP::AmGr1; YFP::AmGr2; YFP::AmGr3) transiently expressed in *Xenopus* oocytes. **(B)** Interaction studies of AmGr1 and AmGr2 by bimolecular fluorescence complementation (BiFC). N- and C-terminal YFP halves fused to either AmGr1 or AmGr2 subunits complemented YFP fluorescence when co-expressed in oocytes, indicating physical interaction, i.e. via homomerization of AmGr1 subunits (YFP^N^::AmGr1 + YFP^C^::AmGr1) or AmGr2 subunits (YFP^N^::AmGr2 + YFP^C^::AmGr2). When corresponding YFP halves were fused to AmGr1 and AmGr2 (YFP^N^::AmGr1 + YFP^C^::AmGr2 / YFP^N^::AmGr2 + YFP^C^::AmGr1) co-expression in oocytes led to yellow fluorescence (YFP complementation), indicating physical interaction of AmGr1 and AmGr2 subunits that assemble to heterotetrameric receptors. N-terminal YFP half fused to AmGr1, co-expressed with C-terminal YFP half fused to AmGr3 (YFP^N^::AmGr1 + YFP^C^::AmGr3), yields no fluorescence, suggesting that heteromer formation does not occur. When corresponding YFP halves fused to AmGr2 and AmGr3 (YFP^N^::AmGr2 + YFP^C^::AmGr3) are co-expressed, fluorescence signals can be detected, indicative of heteromerization. (Scale bar = 200 µm)


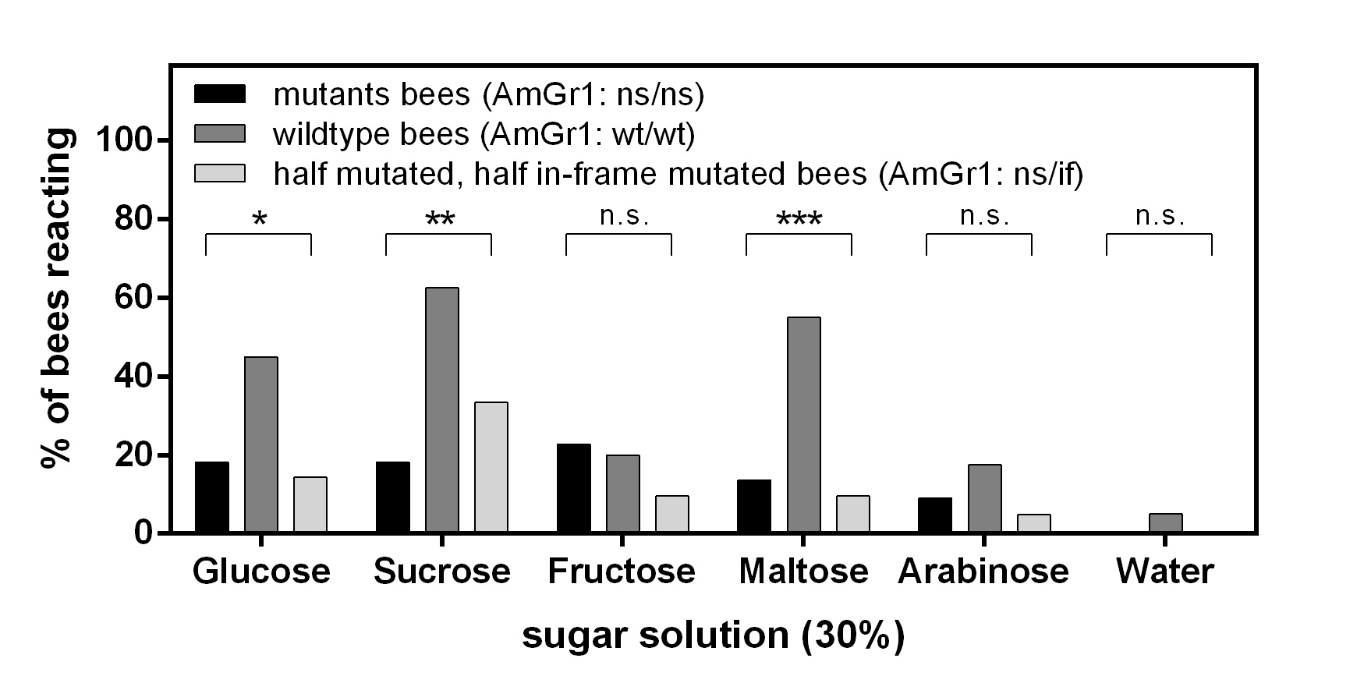


**Figure S4: Behavioural PER test with AmGr1 mutants (ns/ns), half-mutated and half in-frame-mutated bees of AmGr1 (ns/if) and wildtype bees (wt/wt) with a 30% solution of several other sugars (glucose, sucrose, fructose, maltose, arabinose) and water.** The percentage of bees reacting to the representative sugar (only 30% (=0.88 M) solution, only one PER-test) is shown. The respective contingency tables of the three groups (N(ns/ns)=22, N(wt/wt)=40 and N(ns/if)= 21, portioned in reacting and not reacting bees) were analysed with a Chi-Square test and showed, that they were significantly different for glucose (Chi-Square test; Chi^2=^8.199;df=2; p=0.0166; *), sucrose (Chi-Square test; Chi^2=^12.5; df=2; p=0.0019; **) and maltose (Chi-Square test; Chi^2=^17.84; df=2; p=0.0001; ***). In these cases, wildtype bees (wt/wt, dark grey middle bars) reacted with a bigger portion than the AmGr1 mutants (ns/ns, black left bars) or the half-mutated and half in-frame-mutated bees (ns/if, light grey right bars). For the sugars fructose (Chi-Square test; Chi^2=^1.459; df=2; p=0.4822; n.s.) and arabinose (Chi-Square test; Chi^2=^2.356; df=2; p=0.3079, n.s.) and for water (Chi-Square test; Chi^2=^2.203; df=2; p=0.3324; n.s.) there were no significant differences in the three groups. These findings are consistent with the results of the TEVC recordings of additional sugars (Figure S1).

**
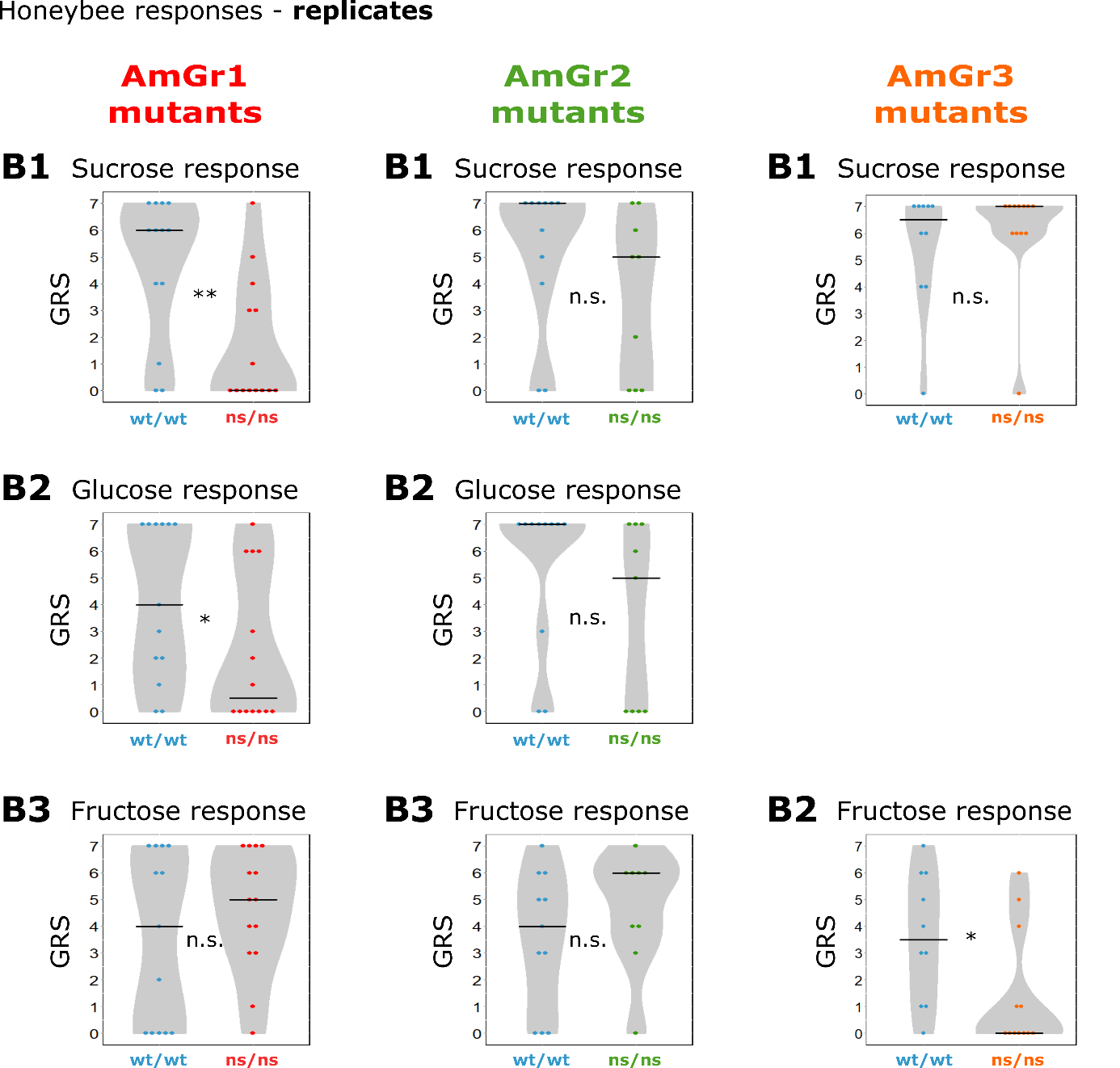
**

**Figure S5: Replicates of behavioural evaluation through proboscis extension response (PER, *in vivo)* according to the section B of each figure (Fig. 1-3).**

AmGr1 mutant bees (ns/ns; N= 14, red) are significantly less responsive towards sucrose (B1) compared to wild-type bees (wt/wt; N=13, blue) (AmGr1 mutants: B1; Mann-Whitney-U, ns/ns vs. wt/wt, p=0.0051, **). Glucose responsiveness (B2) of wild-type bees is significantly higher than those of AmGr1 mutants (AmGr1 mutants: B2; Mann-Whitney-U, ns/ns vs. wt/wt, p=0.0412, *). Both groups do not differ when comparing their responsiveness towards fructose (AmGr1 mutants: B3; Mann-Whitney-U, ns/ns vs. wt/wt, p=0.4528, n.s.).

AmGr2 mutant bees (ns/ns; N=9, green) do not differ in their responsiveness towards sucrose (AmGr2 mutants: B1; Mann-Whitney-U, ns/ns vs. wt/wt, p=0.1940, n.s.), glucose (AmGr2 mutants: B1; Mann-Whitney-U, ns/ns vs. wt/wt, p=0.1284, n.s.) or fructose (AmGr2 mutants: B1; Mann-Whitney-U, ns/ns vs. wt/wt, p=0.3099) when compared with wild-type bees (wt/wt; N=11, blue).

AmGr3 mutant bees (ns/ns; N= 12, orange) do not differ in their responsiveness towards sucrose (B1) compared to wild-type bees (wt/wt; N=10, blue) (AmGr3 mutants: B1; Mann-Whitney-U, ns/ns vs. wt/wt, p=0.5383, n.s.). AmGr3 mutants are significantly less responsive to fructose than wildtype bees (AmGr3 mutants: B2; Mann-Whitney-U, ns/ns vs. wt/wt, p=0.0266, *).

**
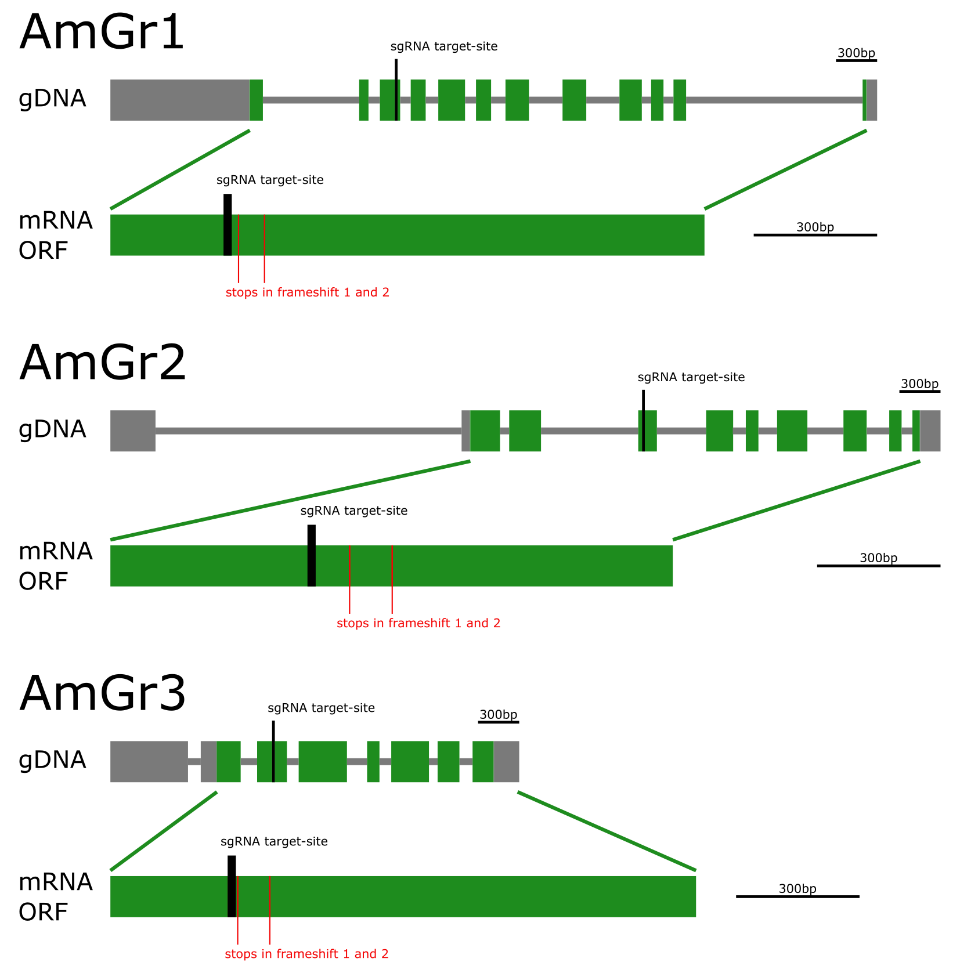
**

**Figure S6: Overview of the receptors (AmGr1, AmGr2 and AmGr3) gene sequences and the early stop codons introduced by mutational frameshifts.**

Target-sites for sgRNAs (see Table S2) were placed in an early exon of the receptor gDNA for AmGr1, AmGr2, and AmGr3. All mutants (and wildtypes) used for the experiments were analyzed by NGS sequencing. Only individuals showing exclusively insertions or deletions (InDels) that were not multiples of three were included as mutants. It was demonstrated that wildtypes were not mutated at the sgRNA target-site. Such InDels shift the triplet code of the mRNAs open reading frame (ORF) and result in an early stop codon (nonsense mutation) in either frameshift 1 or 2 (red). Early stop codons result in non-functional receptor proteins.

**Table S1:** Annotations and primers used for cloning the respective AmGr genes into expression vectors.

| gene | annotation (NCBI) | forward primer (5’-3’) | reverse primer (5’-3’) |
| --- | --- | --- | --- |
| AmGr1 | XM_016912472.2 | GGCTTAAUATGTCGGAACCAGTAGCATT | GGTTTAAUTTACTTCACCTCGCATACAATTG |
| AmGr2 | XM_397125.7 | GGCTTAAUATGCATTCGGAGGATCAAAT | GGTTTAAUTTATTTACCATTAAATTGTAAAAGTAC |
| AmGr3 | XM_016913387.1 | GGCTTAAUATGGAGGTAAAACGAGTAGAAGA | GGTTTAAUTTATTTACCATTAAATTGTAAAAGTAC |

Primers were used to produce a sufficient PCR product from cDNA for cloning the ORF (open reading frame) of the respective gene into an expression vector. The genes listed were subsequently expressed in *Xenopus* oocytes.

**Table S2**: Sequence information for performing CRISRP/Cas9 in the genes of the sugar receptors AmGr1, AmGr2 and AmGr3.

| gene | AmGr1 (sg4) | AmGr2 (sg3) | AmGr3 (sg6) |
| --- | --- | --- | --- |
| crRNA sequence (sgRNA target-site) | 5’-GACTGTTAACCCCCGACACA-3’ | 5’-GGTGTTCTATGGGAACAGTC-3’ | 5’-GCAACTTGTAGTGATGTGCT-3’ |
| Cas9 concentration | 3.13 µM | 3.13 µM | 3.13 µM |
| sgRNA concentration | 23 n/µl (0,5:1) | 92 ng/µl (1:2) | 46 ng/µl (1:1) |
| Injection volume | 400 pl | 400 pl | 400 pl |
| FLA primer forward | 5’-HEX-CATCCATGAGACCAATCATC-3’ | 5’-HEX- CTCTCCATTCGTATTGAAGAGATAC-3’ | 5’-HEX- TGCGTACTTGTATTACTACTTAGTGC-3’ |
| FLA primer reverse | 5’-CGAACTTATCCCAGTTGTGAC-3’ | 5’-GATGATAATTGCGTGAGACATTAC-3’ | 5’-AACAAGTTGCAAATATTTCCAACGG-3’ |
| NGS primer forward | 5’-ACACTCTTTCCCTACACGACGCTCTTCCGATCT XXXXXXXXcatccatgagaccaatcatc-3’ | 5’-ACACTCTTTCCCTACACGACGCTCTTCCGATCTXXXXXXXXctctccattcgtattgaagagatac-3’ | 5’-ACACTCTTTCCCTACACGACGCTCTTCCGATCTXXXXXXXXtgcgtacttgtattactacttagtg-3’ |
| NGS primer reverse | 5’-GACTGGAGTTCAGACGTGTGCTCTTCCGATCTcgaacttatcccagttgtgac-3’ | 5’-GACTGGAGTTCAGACGTGTGCTCTTCCGATCTgatgataattgcgtgagacattac-3’ | 5’-GACTGGAGTTCAGACGTGTGCTCTTCCGATCTGgaaaggagagccaacaatac-3’ |

The table includes the sgRNA target-site and its location related to the whole ORF of the gene (where indels were introduced) and the concentration of the components. To ensure mutations primers for the fluorescence length analysis (FLA) and next generation sequencing (NGS) are listed. Since we used duplex for sequencing „XXXXXXXX” is standing for the tag (5’-CTGTGATG-3’ or 5’-GCGCAATA-3’).
